# The Relationship Between Motivation, Goal Orientation, and Perceived Autonomy Support From the Coach in Young Norwegian Elite Hockey Players

**DOI:** 10.3389/fpsyg.2022.811154

**Published:** 2022-02-16

**Authors:** Arne M. Jakobsen

**Affiliations:** Faculty of Education and Arts, Nord University, Bodø, Norway

**Keywords:** self-determination, ego involvement, task involvement, intrinsic motivation, extrinsic motivation

## Abstract

This study investigates the relationship between motivation, goal orientation, and perceived autonomy support from the coach among junior elite hockey players. The study is based upon the theory of self-determination and the goal orientation theory. The first aim of the study was to investigate whether high scores on task involvement and perceived autonomy support from the coach may explain the intrinsic motivation of the players. Secondly, we sought to discover whether the most autonomous extrinsic motives may be explained by high scores on task involvement and perceived autonomy support from the coach. Lastly, we investigated whether the most controlling extrinsic motives may be explained by greater ego involvement or by both ego and task involvement and less perceived autonomy support from the coach. A total of 401 players aged 14–18 took part in the survey. The results show that intrinsic motivation can be explained by high scores on both task and ego involvement. The two most autonomous extrinsic motives—integrated and identified regulation—were both explained by task and ego involvement and perceived autonomy support from the coach. The two most controlled motives—introjected and external regulation—were both explained by high scores on task and ego involvement.

## Introduction

In this study we have considered the kind of motivation that dominates among junior elite ice hockey players, whether these players are task- or ego-orientated and to what degree the players perceive autonomy support from their coach. We have tried to ascertain whether goal orientation and perceived autonomy support from the coach can explain different kinds of motivation. Possibly which of them is most important? We have not found any other studies which have looked into this relationship with both goal orientation and autonomy support.

Over the last 30 years, numerous studies grounded in self-determination theory have investigated individuals’ motivation in different settings (Deci and Ryan, 1985; Ryan and Deci, 2000, 2019). People may have different reasons for engaging in activities (Vallerand, 2007a,b). When athletes engage in an activity for the satisfaction and pleasure derived from the activity itself, they are intrinsically motivated, whereas behaviors performed to attain material or social rewards are defined as extrinsically motivated (Gillet and Rosnet, 2008; Ryan and Deci, 2017). Intrinsically motivated behavior is associated with satisfaction of three basic psychological needs (Deci and Ryan, 2000): autonomy, competence, and relatedness.

Participation in sport is most often both intrinsically and extrinsically motivated (Lonsdale et al., 2011; Roberts et al., 2018). Intrinsic motivation and more self-determined forms of extrinsic motivation are associated with adaptive emotional, cognitive, and behavioral consequences (Deci and Ryan, 2000). Higher levels of self-determined forms of motivation generally increase chances to succeed and reach the elite level in sports (Martinent and Decret, 2015). Self-determination theory includes two broad classes of non-intrinsic motivation: extrinsic motivation, which is behavior motivated by expected outcomes not inherent in the activity itself, and amotivation, which is a lack of energy directed towards action or intention.

The different external motivational regulations can be differentiated on a motivational continuum based on their relative autonomy (Ryan and Deci, 2007, 2019). The most autonomous external motive is integrated regulation. It reflects a behavior that is close to one’s own values and identity. The next is identified regulation which is an autonomous form of motivational regulation as it reflects to what degree an athlete values sport participation (Sheldon et al., 2017; Roberts et al., 2018). On the motivational continuum, these two autonomous regulations are followed by two less self-determined forms of motivation; introjected and external regulation. These two are often seen as controlled motivational regulations (Ryan and Deci, 2007). Introjected regulation refers to an athlete acting to avoid guilt and shame or to attain ego enhancements, such as pride (Ryan and Deci, 2019). External regulation is the least self-determined form of motivation, and it is characterized by behaviors conducted to satisfy external demands or to reward contingency (Chemolli and Gagné, 2014; Ryan and Deci, 2019). A motivation has been interpreted as a separate construct, outside of the continuum.

A combination of different motivational regulations (self-determined and controlled) may be optimal in achieving high levels of performance depending on the context and the time frame (Vallerand et al., 2008). That is, the quality of motivation of participants in sports and other performance contexts will often reflect a motivational profile based on a combination of self-determined and controlled forms of motivation, also leading to positive outcomes (Roberts et al., 2018).

The actual behavior of coaches in sport would be part of external variables, while the perception of coach’s support of athletes would be more internal variables (Latinjak and Hatzigeorgiadis, 2021). In sport settings, the context is incredibly varied. In Norway, most junior ice hockey coaches work part-time or as volunteers. It has been suggested that the context that coaches are operating in will influence the type of environment they create (Mageau and Vallerand, 2003; Smith et al., 2016). Previous studies have found convergence between observational ratings and perceptual responses on more maladaptive dimensions of coach behavior (Smith et al., 2016). Athletes monitor and pay more attention to negative feedback and are more likely to report when this happens. Positive dimensions of leader behavior, are likely to become established over time. As a result, individuals may pay less attention to such positive behaviors thereby relying on more general reports of the environment (Smith et al., 2016).

Social factors have a deep impact on athletes’ motivation. Autonomy-supportive contexts should facilitate self-determined motivation. Athletes motivation in sport context may be influenced by many factors like sport structure and scholarships (Deci and Ryan, 2000). One of the most important factor is coaches behavior (Mageau and Vallerand, 2003; Alvarez et al., 2009) Perceptions of coaching behavior are related to athletes’ motivation (Amarose, 2007; Langdon et al., 2021) Autonomy-supportive coaches acknowledge athletes’ feelings and perspectives and allow them to be involved in the decision-making process, they provide as much choice as possible within specific limits and rules, and they also provide a rationale for tasks, limits, and rules (Hein and Jöesaar, 2015). Furthermore, autonomy-supportive coaches acknowledge athletes feelings, give them opportunities to take initiative and do independent work, and prevent ego involvement from taking place. These behaviors together represent the autonomy-supportive interpersonal style (Mageau and Vallerand, 2003; Chu et al., 2021). Results from several studies reveal that coaches’ controlling behaviors undermine athletes’ self-determined motivation, while autonomy-supportive behaviors promote it (Amarose and Anderson-Butcher, 2007; Gillet et al., 2010; Behzadnia et al., 2019).

People are driven to achieve for different reasons. According to achievement goal theory, the reasons why they strive to achieve relate to our standards for judging our own competence (Dweck and Leggett, 1988; Pintrich, 2000; Jaakkola et al., 2015). They orient themselves towards meeting these competence standards. If people endorse mastery goals, they are concerned with learning, growth, or understanding. In a sense, they are immersed in the achievement task itself and preoccupied with their own expertise in the domain. If they endorse performance goals, they are concerned with achievement as this relates to others, or how competent they appear to others (Kaplan et al., 2002; Moran and Toner, 2017). Task orientation is focused on self-referenced mastery or improvement in relation to one’s standards. Success is perceived as having occurred when learning, improvement, and mastery are achieved (Williams, 1994). Task-involved athletes are concerned with achieving individual mastery trough the training, and they believe greater success comes with greater effort (Duda and Pensgaard, 2002; Wang et al., 2010). Ego-orientated athletes are concerned with gaining positive judgments from others and comparing their performance to that of competitors (Nicholls, 1989). Ego involvement is a motivational frame of mind in which athletes are concerned with assessing ability. This happens in comparison to others. In this motivational state, athletes are not focused on individual mastery of the task. Rather, they are focused on how they perform in relation to their competitors (Moran and Toner, 2017). Achievement goal theory assumes that goal orientations are not bi-polar opposites of the same construct but independent of each other. This means that an individual can be high and/or low in both orientations at any given time (Nicholls, 1989; Moran and Toner, 2017).

In psychology, the theory refer to ego- and task-orientated athletes. Ego-orientated athletes who rate their ability as inferior to that of competitors are vulnerable to somatic and cognitive anxiety before and during performance (Duda and Nicholls, 1992; Duda and Hall, 1998). People are more likely to drop out of competitions, set standards for their performance that are unrealistically high or low and rate competitions or evaluations as unimportant if they have low estimates of their ability and are ego-orientated (Duda and Nicholls, 1992). Ego-orientated athletes are associated with pressure from coaches and parents to reach exacting goals and with concerns about making mistakes. Task-orientated athletes may also set exacting goals, but these goals conform to the athlete’s own standard (Dunn et al., 2002; Hodge and Gucciardi, 2015; Jakobsen, 2021). Ego-orientated athletes are also more likely to view ability as fixed (Donovan and Williams, 2003).

Athletes who are task-orientated are less vulnerable to somatic and cognitive anxiety (Hall and Kerr, 1997). They have more control over factors that lead to failure and success; this also contributes to heightened enjoyment (Duda and Hall, 2001; Roberts et al., 2007; McCarthy et al., 2008) and intrinsic interest in sport (Duda and Hall, 2001; Roberts et al., 2007).

Many athletes do have multiple goal orientations. Optimal performance may result from endorsement of moderate to high levels of ego and task orientation (Barron and Harackiewicz, 2001; Burton et al., 2011). Top ten athletes in major track and field championships have often been driven by both ego and task goals (Mallet and Hanrahan, 2004). High task orientation may buffer the negative effects of high ego orientations (Hodge and Petlichkoff, 2000; Burton et al., 2011; Moran and Toner, 2017). British elite adolescent athletes with moderate ego/higher task goal orientations use more self-talk than do athletes with higher ego/lower task and moderate task/lower ego goal orientations (Harwood et al., 2003).

In this article, we have considered the kind of motivation that dominates among young elite ice hockey players, whether these players are task- or ego-orientated and to what degree the players perceive autonomy support from their coach. We have tried to ascertain whether goal orientation and perceived autonomy support from the coach can explain different kinds of motivation.

*Hypothesis 1:* Intrinsic motivation will be explained by high scores on task involvement and perceived autonomy support from the coach.

*Hypothesis 2:* The most autonomous extrinsic (integrated. Regulation and identified regulation) motives will be explained by high scores on task involvement and perceived autonomy support from the coach.

*Hypothesis 3:* The most controlling extrinsic motives (introjected regulation and external regulation) will be explained by more ego involvement or both ego and task involvement and less perceived autonomy support from the coach.

## Materials and Methods

### Participants

The participants consisted of 401 young Norwegian ice hockey players 14–18 years of age. Players were told that participation in the survey was voluntary, and they were free to withdraw at any time. No players refused to take part. Of these, 49% were 15 and 16 years old. Of the players, 94 were representing Norwegian national teams for U-20 and U-18. Only 59 players were also competing in a sport outside of ice hockey. There were 59 goalkeepers, 127 defenders, 201 forwards, and 19 who play both forward and defender. We obtained parental consent to participate in the study. The study is approved by the Norwegian Centre for research data.

### Measures

The data was collected during a one-week preseason junior elite hockey camp arranged by the Norwegian Hockey Federation. The players had 1 h to complete the questionnaires. The questionnaires were on paper, and the author has filtered the responses. The author was present during the completion. The author thus had the opportunity to resolve any ambiguities regarding the questions. Unanswered questions were deleted.

All questionnaires were translated into Norwegian and back to English. They were also tested on a group of students for validation.

We used the Sport Motivation Scale questionnaire (Pelletier et al., 2013) to measure motivation among the players. This is a revised version of the SMS (Pelletier et al., 1995). It contains 18 questions and six factors, representing intrinsic regulation (*α* = 0.65) integrated regulation (*α* = 0.66), identified regulation (*α* = 0.72), introjected regulation (*α* = 0.57), external regulation (*α* = 0.64), and amotivation (*α* = 0.64). Only identified regulation was acceptable, introjected regulation was poor, and the other factors yielded questionable reliability values (Cronbach’s *α*; Lavrakas, 2008). We did also run a confirmatory factor analysis with an acceptable result (Hair et al., 2018). Indices obtained (GFI = 0.87, AGFI = 0.83, RMSEA = 0.06, CFI = 0.93) suggested the appropriate fit for the model with the data. Each factor is represented in three questions. The players were asked why they play ice hockey and were asked to answer on a seven-point Likert-type scale (where 1 = does not agree at all and 7 = completely agrees).

To examine perceived coach autonomy support, we used the short version of the Sport Climate Questionnaire (Deci and Ryan, 2016) with six items worded in terms of “my coach” (*α* = 0.82, good; Lavrakas, 2008). In addition, indices obtained from confirmatory factor analysis (GFI = 0.94, AGFI = 0.91, RMSEA = 0.08, CFI = 0.95) indicated the appropriate fit of the model with the data They were answered on a Likert-type scale of 1 to 7 (where 1 = does not agree at all and 7 = completely agrees). High average scores represent a high level of perceived autonomy support.

To measure dispositional goal orientation, we used the Task and Ego Orientation in Sport Questionnaire (TEOSQ; Duda, 1989; Duda and Hall, 1998). The TEOSQ has a two-factor structure, representing task (seven items, *α* = 0.82) and ego (six items, *α* = 0.87) orientations. In addition, indices obtained from confirmatory factor analysis (GFI = 0.92, AGFI = 0.88, RMSEA = 0.07, CFI = 0.94) indicated the appropriate fit of the model with the data. Given that the questionnaires were administered in an ice hockey context, the players were encouraged to think about how successful they felt in relation to their team and then indicate on a five-point Likert-type scale (where 1 = strongly disagree and 5 = strongly agree) whether they agreed or disagreed with the items reflecting a task orientation (e.g., “I feel successful when I work really hard”) or ego orientation (e.g., “I feel successful when others cannot do as well as I can”).

### Statistical Analyses

IBM SPSS Statistics 26 was used for the calculation. Descriptive statistics, means, and standard deviations were obtained for all variables. Correlations were calculated to test relationships among all variables. Five different regression analyses were conducted where task and ego involvement on the one hand and, on the other, perceived autonomy support from the coach are the independent variables. The dependent variables are: (1) intrinsic motivation, (2) integrated regulation, (3) identified regulation, (4) introjected regulation, and (5) external regulation (Hair et al., 2018).

## Results

There are high scores for intrinsic motivation (*M* = 5.99) and for the most autonomous extrinsic motives, such as integrated regulation (*M* = 6.0) and identified regulation (*M* = 5.73; Table 1). There is also a high score on task involvement (*M* = 4.52) and a significant difference (sign. <0.01) between task and ego involvement (*M* = 3.01). The score for perceived autonomy support from the coach is close to the median (*M* = 4.52).

**Table 1:** Correlation between all variables.

		1	2	3	4	5	6	7	8	9
1.	Intrinsic motivation									
2.	Integrated regulation	0.61^**^								
3.	Identified regulation	0.56^**^	0.66^**^							
4.	Introjected regulation	0.40^**^	0.55^**^	0.54^**^						
5.	External regulation	0.20^**^	0.24^**^	0.28^**^	0.53^**^					
6.	Amotivation	−0.28^**^	−0.32^**^	−0.16^**^	−0.18^**^	0.06				
7.	Task involvement	0.51^**^	0.48^**^	0.45^**^	0.40^**^	0.14^**^	−0.35^**^			
8.	Ego involvement	0.00	0.13^*^	0.13^*^	0.23^**^	0.28^**^	0.07^**^	0.07		
9.	Autonomy support from the coach	0.26^**^	0.23^**^	0.22^**^	0.06	0.09	−0.13^*^	0.21^**^	0.04	
	N	396	391	395	393	396	394	398	393	390
	*M*	5.99	6.00	5.73	5.34	4.43	1.55	4.52	3.01	4.73
	*SD*	0.97	0.91	1.06	1.19	1.38	0.82	0.50	0.92	1.34
	*α*	0.65	0.66	0.72	0.57	0.64	0.64	0.82	0.87	0.82

*Participants (N), mean (M), standard deviation (SD) and alpha values (α) for the variables*.

* <0.05;

** <0.01.

Task and ego involvement are broken down into a dichotomous variable that contains high and low, with the split at 3.0.

There are 191 players who are high on task involvement and low on ego involvement. This is almost the same figure as for those who are high on both (200; Table 2).

**Table 2:** Cross tabulation of task and ego involvement with % of total.

			Ego involvement		Total
			Low	High	
Task involvement	Low		5	1	6
		% of Total	1.3%	0.3%	1.5%
	High		186	199	385
		% of Total	47.6%	50.9%	98.5%
Total			191	200	391
		% of Total	48.8%	51.2%	100.0%

The first regression analysis had intrinsic motivation as a dependent variable. Task involvement and perceived autonomy support from the coach explained 28% of the dependent variable (sign = 0.01). Task involvement alone explained 26% (Table 3).

**Table 3:** Regression analysis with intrinsic motivation, integrated-, identified-, introjected- and extrinsic regulation as the dependent variables, and task- and ego involvement and perceived autonomy support from the coach as the independent variables.

Independent variables	Dependent variables					
	Intrinsic motivation	Integrated regulation	Identified regulation	Introjected regulation	Extrinsic regulation
	Task involvement (β)	0.48^**^	0.44^**^	0.42^**^	0.40^**^
0.11^*^		Ego involvement (β)	−0.04	0.09	0.09^*^
0.20^**^	0.28^**^		Perceived autonomy from the coach (β)	0.15^**^	0.13^**^
0.12^*^	−0.04	0.04		*R* ^2^	0.28
0.24	0.22	0.20	0.09		*F*	50.00^**^	40.68^**^	35.94^**^	32.10^**^	13.36^**^	

* <0.05;

** <0.01.

In the second analysis, the dependent variable—integrated regulation—was explained by both task involvement (*R*^2^ = 0.24) and perceived autonomy support from the coach (*R*^2^ change = 0.02). Both are significant at the 1% level.

In the third analysis, task involvement (sign = 0.01), perceived autonomy support from the coach (sign = 0.05) and ego involvement (sign = 0.05) all explain 22% of the dependent variable identified regulation (Table 3). The most important independent variable is task involvement, which on its own explains 20% of the dependent variable.

When we used introjected regulation as the dependent variable, task and ego involvement yielded *R*^2^ = 0.20 (*R*^2^ change = 0.04). Both were significant at 1%. Task involvement explained on its own 16% of the dependent variable.

Finally, we looked at an analysis where external regulation was the dependent variable. Here both task (sign = 0.05) and ego (sign = 0.01) involvement were the explanation, but only had *R*^2^ = 0.90. Ego involvement alone explained 8% of the variable.

## Discussion

The purpose of this study was to consider what kind of motivation that dominates among junior elite ice hockey players, whether these players are task- or ego-orientated and to what degree the players perceive autonomy support from their coach. We also tried to ascertain whether goal orientation and perceived autonomy support from the coach can explain different kinds of motivation.

As expected, the players had a high score on intrinsic motivation and on the most autonomous extrinsic motives (Ryan and Deci, 2000, 2017; Standage et al., 2019). More surprisingly, they also had a high score on introjected regulation and a relatively high score on extrinsic regulation, which are more controlling motives. This can be explained by people typically having multiple motives, both intrinsic and extrinsic, for engaging in sport (Hagger and Chatzisarantis, 2008; Lonsdale et al., 2011; Hancox et al., 2018). Earlier research confirms that sustained exercise is most likely when a person has both well-internalized extrinsic motivation and intrinsic motivation (Duncan et al., 2010; Vlachopoulos et al., 2010; Smith et al., 2011).

Furthermore, we see that half of the players had a low score on ego involvement and a high score on task involvement. The other half had high scores on both ego and task involvement. This is not unusual. We know that many athletes have multiple goal orientations and that high task orientation may buffer the negative effects of high ego orientations (Barron and Harackiewicz, 2001; Burton et al., 2011; Moran and Toner, 2017).

Perceived autonomy support from the coach yields a mean of 4.73. This is not a very high score. Nearly 40% of the players rated their coach on a scale of one to four. As we know that perceived autonomy support from coaches positively predicts relatedness, autonomy, competence need satisfaction and intrinsic motivation (Edmunds et al., 2007; Langdon et al., 2021), this result is not good for the reputation of Norwegian junior hockey coaches.

Our first hypothesis predicted that intrinsic motivation would be explained by high scores on task involvement and perceived autonomy support from the coach (Edmunds et al., 2007; Stanley et al., 2012; Benish and Langdon, 2021). These two independent variables did explain 28% of the dependent variable. This means that our hypothesis is confirmed. Task-involved athletes are focused on mastery goals and are concerned with learning, growth, or understanding. In a sense, they are immersed in the achievement task itself and preoccupied with their own expertise in the domain (Kaplan et al., 2002; Moran and Toner, 2017). These athletes are focused on self-referenced mastery or improvement in relation to one’s standards. Success is perceived as having occurred when learning, improvement, and mastery are achieved (Williams, 1994). Task-involved athletes are concerned with achieving individual mastery trough the training, and they believe greater success comes with greater effort (Duda and Pensgaard, 2002; Wang et al., 2010). Perceptions of coaching behavior are also related to athletes’ motivation (Amarose, 2007; Langdon et al., 2021) Autonomy-supportive coaches acknowledge athletes’ feelings and perspectives and allow them to be involved in the decision-making process, they provide as much choice as possible within specific limits and rules, they also provide a rationale for tasks, limits, and rules (Hein and Jöesaar, 2015) Their intrinsic motivation will increase (Langdon et al., 2021).

Secondly, we predicted that the most autonomous extrinsic (integrated regulation and identified regulation) motives would be explained by high scores on task involvement and perceived autonomy support from the coach (Chatzisarantis et al., 2007; Edmunds et al., 2007; Behzadnia et al., 2019). This prediction is confirmed. 22% of the dependent variable integrated regulation was explained by task involvement, as expected. The remainder (2%) was explained by perceived autonomy support from the coach.

Identified regulation, which is also an autonomous motive, was explained by all three independent variables, with task involvement being the most important variable. In conclusion, we can retain our hypothesis. These findings support that participation in sport is both intrinsically and extrinsically motivated (Lonsdale et al., 2011; Roberts et al., 2018). A good result for the Norwegian ice hockey federation is that higher levels of self-determined forms of motivation generally increase chances to succeed and reach the elite level in sports (Martinent and Decret, 2015).

Lastly, we looked at which of the three independent variables would best explain the two most controlling motives (introjected and external regulation) in the self-determination theory. The expectation was that more ego involvement, or both ego and task involvement, together with less perceived autonomy support from the coach, would explain the dependent variables (Edmunds et al., 2007; Wilson et al., 2008; Stanley et al., 2012; Stuart, 2013; Chu et al., 2021). Both dependent variables were explained by task and ego involvement. 20% of introjected regulation was accounted for mostly by task involvement (16%). Task involvement explained 1% of external regulation, and 8% were explained of ego involvement. As expected, perceived autonomy support from the coach did not explain either of the two most controlling motives (Mageau and Vallerand, 2003; Chu et al., 2021). We can retain our hypothesis. Ego-orientated athletes who rate their ability as inferior to that of competitors are more likely to drop out of competitions, set standards for their performance that are unrealistically high or low and rate competitions or evaluations as unimportant (Duda and Nicholls, 1992). Ego-orientated athletes are associated with pressure from coaches and parents to reach exacting goals, and with concerns about making mistakes (Hodge and Gucciardi, 2015; Jakobsen, 2021), it is also more likely that they view ability as fixed (Donovan and Williams, 2003). As we see, they do not have a perception of autonomy support from their coach.

## Conclusion

Among Norwegian junior elite ice hockey players, both intrinsic and the most autonomous extrinsic motives are dominant. More or less all of the players have a task involvement with their sport, and nearly 50% are both task and ego involved.

Most players perceive a relatively high level of autonomy support from their coach, even if nearly 40% have a medium or low score on this parameter.

Intrinsic motivation is explained by high scores on both task and perceived autonomy support from the coach, where task involvement is more important.

The two most autonomous extrinsic motives (integrated and identified regulation) were both explained by task and perceived autonomy support from the coach. Here, too, task involvement dominated. In addition, ego involvement also had an explanation on identified regulation (less than 1%), but not on integrated regulation.

Finally, we found that the two most controlled motives, introjected and external regulation, were both explained by high scores on task and ego involvement. Task involvement was the most important explanation on introjected regulation. External regulation was mostly explained by a high score on ego involvement.

Based upon the following study practical recommendations for coaches will be:

We found out that intrinsic motivation and the most autonomous form of extrinsic motivation are explained by task-involved players which perceives high autonomy support from their coaches. We also found out that ego involvement explained mostly external regulation which are the most controlling form of external motivation. This means that the coaches should encourage and create task-involved athletes which are concerned with achieving individual mastery trough the training, and believes greater success comes with greater effort. The coaches should try to avoid making ego-oriented players (Duda and Pensgaard, 2002; Wang et al., 2010). This is probably the most important thing to do.

The coaches should also be autonomy-supportive to their players by giving them acknowledgment for their feelings, give them opportunities to take initiative and do independent work, and prevent ego involvement from taking place. These behaviors together represent the autonomy-supportive interpersonal style (Mageau and Vallerand, 2003; Chu et al., 2021). Here it should be taken into account that the study was done in a Norwegian context. Norwegian players may to a greater extent be brought up in a more task-oriented environment and have a greater expectation of autonomy support by the coach than players brought up in a different cultural context.

There might be some other limitations in the study as well. Data collected preseason may be affected by the players forgetting what the situation was. The players’ response may also have been affected by the context, for instance, the author’s and other players presence. The questionnaires are translated into Norwegian and validated with the help of students who are older than the representative players and may thus have influenced the understanding of the questions. This may explain the low alpha values of some variables. The samples of the studies were recruited Norwegian players. Keeping in view the scope of this study, the samples were adequate. However, for future research it would be beneficial to include samples from other countries so as to increase its generalizability and external validity. Lastly, we could have used more advanced statistical methods like structural equation modeling for the analysis.

## Data Availability Statement

The raw data supporting the conclusions of this article will be made available by the authors, without undue reservation.

## Ethics Statement

The studies involving human participants were reviewed and approved by Norwegian centre for research data. Written informed consent to participate in this study was provided by the participants’ legal guardian/next of kin.

## Author Contributions

The author confirms being the sole contributor of this work and has approved it for publication.

## Conflict of Interest

The author declares that the research was conducted in the absence of any commercial or financial relationships that could be construed as a potential conflict of interest.

## Publisher’s Note

All claims expressed in this article are solely those of the authors and do not necessarily represent those of their affiliated organizations, or those of the publisher, the editors and the reviewers. Any product that may be evaluated in this article, or claim that may be made by its manufacturer, is not guaranteed or endorsed by the publisher.
